# Territorial Vulnerabilities and Healthcare Capacity During the COVID-19 Pandemic: A Spatiotemporal Study of Hospitalizations and Mortality in a Large Metropolitan Area in Brazil

**DOI:** 10.3390/ijerph23070928

**Published:** 2026-07-20

**Authors:** Ana Maria Viegas, Selma Costa de Sousa, Diogo Tavares Cardoso, Aline Dayrell Ferreira Sales, Isabela Farnezi Veloso, David Soeiro Barbosa

**Affiliations:** 1 Laboratory of the Epidemiology of Infectious and Parasitic Diseases, Department of Parasitology, Institute of Biological Sciences, Federal University of Minas Gerais, Belo Horizonte 31270-901, Brazil; aviegas@ufmg.br (A.M.V.); scs2007@ufmg.br (S.C.d.S.); diogo.tavares0@yahoo.com.br (D.T.C.); 2 Graduate Program in Public Health, School of Medicine, Federal University of Minas Gerais, Belo Horizonte 30130-100, Brazil; dayrell@ufmg.br; 3 Belo Horizonte Urban Health Observatory, School of Medicine, Federal University of Minas Gerais, Belo Horizonte 30130-100, Brazil; 4 Health Department of Contagem, Contagem 32210-110, Brazil; ifveloso@gmail.com; 5 Health Department of Betim, Betim 32600-412, Brazil

**Keywords:** health services accessibility, public health, social vulnerability, health equity

## Abstract

Objective: This study aimed to identify priority areas, social vulnerability, and healthcare capacity and networks while considering hospitalization and mortality rates due to Severe Acute Respiratory Syndrome (SARS) caused by COVID-19 during the public health emergency period. Materials and Methods: A spatiotemporal analysis was performed to assess hospitalization, mortality, and case fatality rates among hospitalized patients due to severe COVID-19 across the municipalities of the Metropolitan Region of Belo Horizonte (MRBH), Brazil, between 2020 and 2022. The findings were contextualized by considering the social vulnerability of the population as well as the healthcare infrastructure and organizational characteristics of municipalities and health regions. Results: Municipalities located in the central area of the MRBH showed higher hospitalization rates, corresponding to areas with a greater concentration of healthcare infrastructure. However, mortality and case fatality rates among hospitalized patients were higher in municipalities with more limited healthcare infrastructure and greater social vulnerability. Conclusion: The spatiotemporal disparities in hospitalization and mortality risks suggest health inequities that seem to be influenced by social determinants and healthcare system vulnerabilities. These findings emphasize the imperative for public health emergency responses to prioritize the strengthening of health system organization and the establishment of effective care pathways to enhance access and service delivery, particularly in municipalities exhibiting higher levels of social vulnerability.

## 1. Introduction

Public Health Emergencies (PHEs) are situations that require measures to prevent, control, and contain risks, damage, and harm to public health. These conditions include epidemiological situations such as disease outbreaks and epidemics, as well as disasters that limit the care that can be provided to the general population [1]. A health risk situation is considered to occur when epidemiological data demonstrate the potential risk of a PHE, along with information regarding the installed capacity of the healthcare network that shows the possibility of health services being overloaded, situations of natural or technological disasters, and climate crises [2].

The PHE of the COVID-19 pandemic generated a great demand for diagnoses and care, challenging the health system and, in turn, requiring reorganization of the hospital and outpatient network to care for those affected by the disease and to diminish the case fatality rate among hospitalized patients. This reorganization presented a challenge concerning the local specificities of population density and rearrangements of regional health departments, such as in the Metropolitan Region of Belo Horizonte (MRBH).

The MRBH is the third largest urban agglomeration in Brazil (population of 5,893,004 inhabitants) [3], and is considered a rural–urban fringe, with 1.89% of the population residing in rural areas [4]. The region features industrial activity and zones with elevated levels of particulate matter pollution [5]. Located in the central region of Minas Gerais [6], it has 50 municipalities with a large circulation of people [3]. The geographic coordinates of Belo Horizonte, the largest city in the region, are latitude 19.9245° S and longitude 43.9352° W [7]. The agglomeration of people and the mobility between municipalities from large cities to their outskirts favored the dispersion of COVID-19 and the spread of the disease [8].

The state of Minas Gerais has health regions with an infrastructure that includes hospital beds and admissions [9]. The health regional offices provide services and beds according to local availability. There are procedures that require the transfer of patients to other health regional offices. During the pandemic, each health region organized the deployment of hospital beds and health services according to the population’s needs [9]. The Metropolitan Region of Belo Horizonte (MRBH) is composed of nine health microregions. The number of general hospitals in each region is as follows: Belo Horizonte, 75; Betim, 7; Contagem, 7; Itabira, 2; Itaúna, 2; Pará de Minas, 1; Sete Lagoas, 4; and Vespasiano, 6 [9].

COVID-19 patients sometimes progressed to Severe Acute Respiratory Syndrome (SARS), hospitalization and, in some cases, intensive care [10]. Severe COVID-19 cases were defined based on the guidelines established by the Brazilian Ministry of Health, which served as the standard for notification and classification across the country, including the state of Minas Gerais. According to these guidelines, severity is defined by the presence of respiratory distress, oxygen saturation below 95% in ambient air, respiratory rate ≥ 24 breaths per minute, imaging evidence of pneumonia (especially bilateral infiltrates), the need for supplemental oxygen or ventilatory support, and/or hemodynamic instability requiring vasopressor support [10]. While case definitions were standardized, hospital and ICU admission criteria showed variation across municipalities due to bed availability, healthcare infrastructure differences [11] between rural and urban areas, and local protocols that incorporated additional screening criteria based on comorbidities and age. With the introduction of vaccination in January 2021, there was a reduction in hospitalizations and mortality. However, the emergence of the new SARS-CoV-2 variants Delta and Omicron did not reduce morbidity [12].

After the pandemic and the challenges faced by the health system, it became imperative to identify locations with the highest risk of hospitalization and deaths and to reflect on which factors had the greatest impact on the occurrence of unfavorable health outcomes, especially from the perspective of social vulnerability and the organization of the care network. Understanding this is essential in order to propose effective strategies for future PHEs that aim to expand access to health services and reduce mortality and inequities in access between municipalities.

This study sought to identify priority areas, considering social vulnerability and health networks, through spatial and spatiotemporal analyses of hospitalization and death rates in PHEs in the municipalities of the MRBH from 2020 to 2022.

## 2. Materials and Methods

### 2.1. Study Design and Data Sources

This work was an ecological study, with data extracted from the Influenza Epidemiological Surveillance Information System (SIVEP-Gripe), made available by the Health Department of the state of Minas Gerais, and general population data from the Brazilian Institute of Geography and Statistics (IBGE) CENSUS 2023.

SIVEP-Gripe is an information system used to monitor and record cases of severe COVID-19, including surveillance for respiratory viruses such as influenza and coronavirus (SARS-CoV-2), and consists of secondary data from public and private health establishments. Data quality assurance procedures were implemented, comprising duplicate elimination, temporal data validation, and categorical variable standardization.

This study was conducted in accordance with the ethical guidelines of the Brazilian National Health Council (Resolution No. 510/2016), which regulates research involving secondary and anonymized data. It was approved by the Research Ethics Committee of the Universidade Federal de Minas Gerais under CAAE 55149621.2.0000.5149.

### 2.2. Study Area

**Figure 1:**
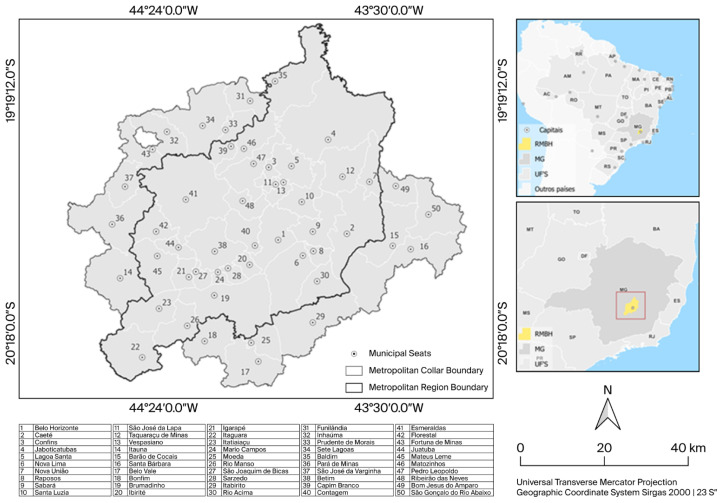
Location of Minas Gerais, Brazil. Metropolitan Region of Belo Horizonte, metropolitan collar, and division between areas with the identification of municipalities.

The MRBH was selected for this study as it is the most populous region in the state of Minas Gerais [3]; it has a metropolitan collar and core, organized into two macroregions and seven health microregions for referrals for hospitalization and healthcare [6] (Figure 1).

### 2.3. Outcome Variables

Data on hospitalizations and deaths due to severe COVID-19, available in the SIVEP-Gripe database of the Health Department of the state of Minas Gerais, were analyzed, and from these data, the hospitalization rates, case fatality rate of hospitalized patients, and mortality rates per year were calculated and aggregated by municipalities of residence, considering the geocode of each municipality.

The hospitalization rate was calculated by dividing the number of hospitalizations due to severe COVID-19 of residents of each municipality in the years 2020, 2021, and 2022 by the population of each municipality and multiplying by 10,000. The mortality rate was calculated by dividing the number of deaths due to severe COVID-19 per municipality by the population of each municipality and multiplying by 100,000. To calculate these rates, the 2022 Census/IBGE population count was employed. The case fatality rate among hospitalized patients was calculated by dividing the number of deaths due to severe COVID-19 by the number of hospitalizations due to severe COVID-19 per year of occurrence multiplied by 100.

### 2.4. Spatial and Spatiotemporal Analyses

For statistical analysis of spatial dependence, the Moran Autocorrelation Index was used, calculating both the Global Moran Index, which informs the level of spatial interdependence between the study areas (polygons), and the Local Moran Index, which analyzes the covariance between a given polygon and a neighborhood defined as a function of a distance in order to investigate the patterns of spatial dependence at the local level. To verify dependence between the areas, a first-order neighborhood matrix (Queen) was created, considering neighboring areas as bordering areas. The Global Moran Index analyzed the spatial distribution pattern of each variable by municipality. The values of the Global Moran Index range from −1 to 1, with positive values indicating positive autocorrelation, negative values indicating negative autocorrelation, and values close to zero indicating no correlation. The Local Moran Index tests local autocorrelation and detects spatial objects influenced by the Global Moran Index, giving the value of the unit of analysis, whether it is related to its neighbors, and whether positive values are present. Thus, the Moran map shows four quadrants, where quadrants Q1 and Q2 indicate clusters with high–high (AA) and low–low (BB) positive autocorrelation. Our study verified the presence or absence of spatial dependence and the existence of spatial clusters. To visualize clusters of municipalities, Moran maps were created, which present statistically significant results (*p* < 0.05 and 95% CI) for the absolute number of hospitalizations, hospitalization rates, the case fatality rates of hospitalized patients, and mortality rates for severe COVID-19 cases in the period from 2020 to 2022. The Global and Local Moran Indices [13] were calculated and constructed using GeoDa Software, version 1.10.

Spatiotemporal analysis was used to define epidemiological risk. This is a technique that enables analysis through the gradual scanning of spatiotemporal information, indicating the number of observed and expected events within each location. The statistical scanning analysis centers circles in each location and compares the proportion of events (hospitalization rate, case fatality rate of hospitalized patients, and mortality rate) both inside and outside of these. Spatial clusters were identified using the discrete Poisson model, which is suitable for analyzing count data, such as the number of hospitalizations and deaths, which are discrete events occurring within a defined population over a specific period. To calculate the scanning statistics, the following parameters were used: 1. no geographic overlap of clusters; 2. a maximum spatial cluster size set at 50% of the exposed population to allow the detection of both small, localized outbreaks and broader regional patterns within the diverse metropolitan area; 3. the maximum temporal clusters were also set to 50% of the study period to capture both the short-term peaks and longer waves of the pandemic. A temporal precision of one year was applied to align with annual data variations and trends. Circular scanning windows were used to ensure methodological neutrality, and 999 Monte Carlo replications were performed to ensure robust significance testing [14]. We chose to maintain these default settings to avoid arbitrary parameterization. In the context of a densely populated and heterogeneous metropolitan area, these choices allowed the method to detect clusters across a wide range of scales while maintaining a balance between sensitivity and specificity.

Spatial clusters were identified using the discrete Poisson model. This model is particularly appropriate for analyzing count data, such as the number of hospitalizations and deaths, which are discrete events occurring within a defined population over a specific period. To calculate the spatial scan statistics, the following settings were used: no occurrence of geographic overlap of clusters; clusters with a maximum size equal to 50% of the exposed population (chosen to identify both small, localized outbreaks and broader regional patterns within the diverse metropolitan area); maximum size of temporal clusters equal to 50% of the study period (allowing for the detection of clusters spanning various durations, from short-term peaks to longer waves of the pandemic); and standardized temporal precision of one year (to align with annual data variations and trends), with circular sets (a standard, unbiased scanning window shape) and 999 repetitions (to ensure robust statistical significance testing through Monte Carlo simulations) [14]. We chose to use the default mode settings in the analysis so that the choice is not arbitrary, and this parameterization allowed SaTScan to effectively evaluate clusters of varying scales, from very small to very large, thereby balancing detection sensitivity and specificity within the high population density of the study area.

This model considered the space and time in which hospitalizations, mortality, and fatality occurred. Clusters with *p* ≤ 0.05 were considered significant. The technique was configured to identify high-risk and low-risk clusters. To compare the areas, the program presented the relative risk (RR) of each cluster. To identify spatiotemporal clusters, scan statistics were applied using SaTScan software, version 9.4.4 [14], and maps were constructed in QGIS software, version 3.10.

## 3. Results

A total of 67,091 hospitalizations due to severe COVID-19 were recorded from 2020 to 2022, with the highest number of hospitalizations occurring in the capital of Minas Gerais, Belo Horizonte (*n* = 34,894, 52.0%). In the MRBH, the hospitalization rates ranged from 40.51/10,000 inhabitants (Nova União) to 146.91/10,000 inhabitants (Belo Horizonte). Mortality rates ranged from 1.00/100,000 inhabitants (Fortuna de Minas) to 4.36/100,000 inhabitants (Mário Campos). Finally, the case fatality rate among hospitalized patients ranged from 5.28% (Baldim) to 2.13% (Belo Vale).

### 3.1. Spatial Autocorrelation of Hospitalizations, Deaths, Hospitalization Rates, Mortality Rates, and Case Fatality Among Patients Hospitalized for Severe COVID-19

**Figure 2:**
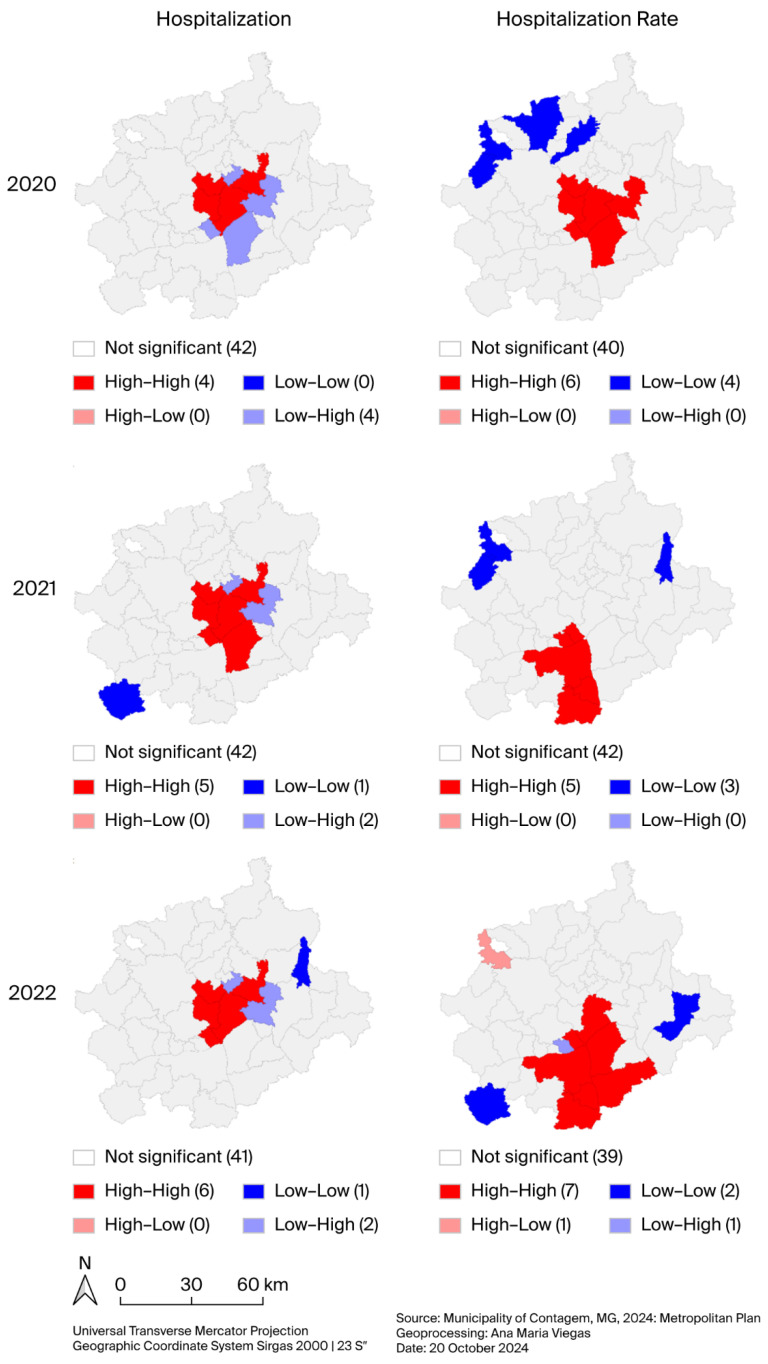
Moran maps of the absolute number of hospitalizations and hospital admission rates for severe COVID-19 in the municipalities of the MRBH with high, medium, and low significance, 2020 to 2022.

Positive spatial autocorrelation and spatial dependence were observed in the years 2020, 2021, and 2022 for the absolute number of hospitalizations (I = 0.094, I = 0.115, I = 0.094), hospital admission rates (I = 0.394, I = 0.302, I = 0.308), absolute number of deaths (I = 0.118, I = 0.141 and I = 0.091), mortality rates (I = 0.138, I = 0.078, I = 0.086), and the case fatality rate among hospitalized patients (I = 0.172 in 2021) due to severe COVID-19 in the MRBH. In the Moran maps, statistically significant clusters of municipalities stood out (*p* < 0.05) (Figure 2). In 2020, for the absolute number of hospitalizations due to severe COVID-19, a high-high cluster with four municipalities was observed comprising Belo Horizonte, Contagem, Ribeirão das Neves, and Santa Luzia; this cluster expanded with the addition of the municipality of Ibirité in 2021 and Nova Lima in 2022 (a cluster of six municipalities).

Regarding the hospitalization rate, in 2020 a high–high pattern was observed in a cluster of six municipalities: Belo Horizonte, Contagem, Ibirité, Nova Lima, Ribeirão das Neves, and Sabará. This pattern was observed in a cluster with five municipalities (Ibirité, Sarzedo, Brumadinho, Moeda, and Belo Vale) in 2021 and in a cluster with seven municipalities (Belo Horizonte, Ibirité, Brumadinho, Nova Lima, Moeda, Belo Vale, and Itabirito) in 2022 (Figure 2).

**Figure 3:**
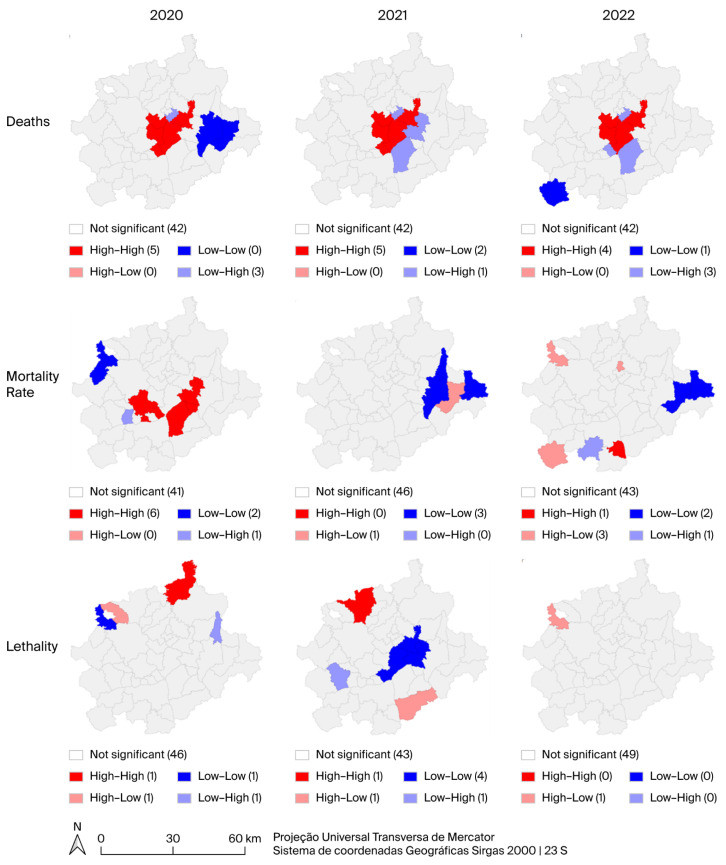
Moran maps of deaths, mortality rates, and case fatality rates among those hospitalized due to severe COVID-19 from 2020 to 2022 in municipalities of the MRBH with high, medium, and low significance.

In 2020 and 2021, a high–high pattern was observed for death outcome in a cluster with five municipalities: Belo Horizonte, Contagem, Ibirité, Ribeirão das Neves, and Santa Luzia. In 2022, this pattern was found in a cluster with four municipalities: Belo Horizonte, Contagem, Ribeirão das Neves, and Santa Luzia. For mortality rates, in 2020, the high–high pattern was observed in a cluster with six municipalities (Betim, Ibirité, Mário Campos, Nova Lima, Raposos, and Sabará), while in 2022 it was observed in the Moeda cluster (Figure 3). The case fatality rate among hospitalized patients showed a high–high pattern in the Baldim cluster in 2020 and in the Sete Lagoas cluster in 2021; however, this pattern was not evident in 2022 (Figure 3).

### 3.2. Spatiotemporal Scan Analysis

**Figure 4:**
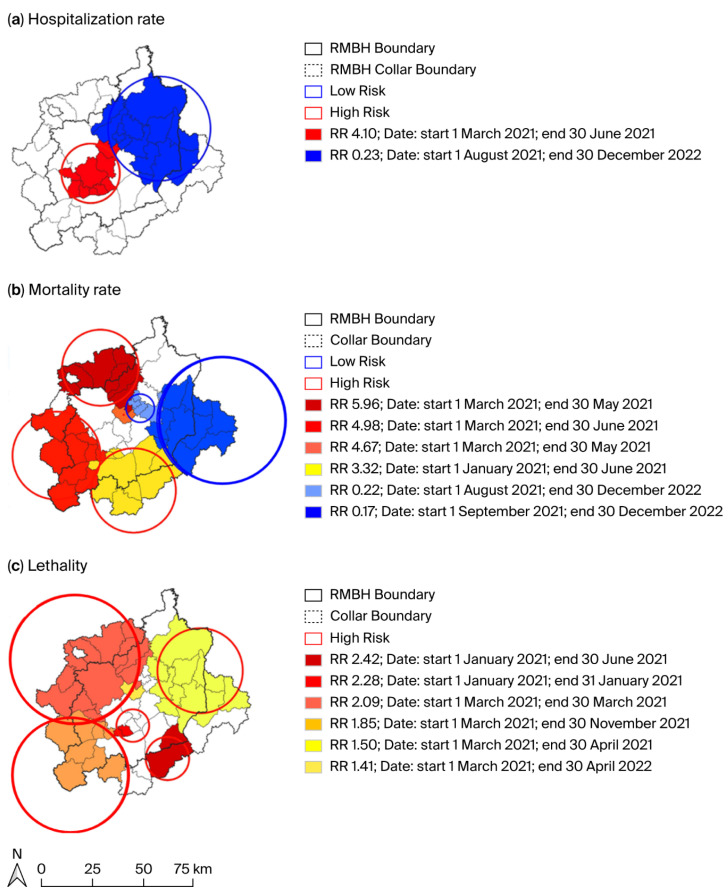
Spatiotemporal permutation analysis of the hospitalization rate (**a**), mortality rate (**b**), and case fatality rate among hospitalized patients (**c**) due to severe COVID-19 in the MRBH, 2020 to 2022.

In the analysis of the spatiotemporal risk scan (*p* ≤ 0.05) for hospitalization rates in 2021, a cluster with a higher risk of hospitalization (RR = 4.10) was identified (Betim, Contagem, Ibirité, Igarapé, Mário Campos, Juatuba, Sarzedo, Ribeirão das Neves and São Joaquim de Bicas) (Figure 4a).

The spatiotemporal scan analysis for mortality rates due to severe COVID-19 detected a high-risk cluster (RR = 5.96) (Sete Lagoas, Fortuna de Minas, Inhaúma, Funilândia, Prudente de Morais, Matozinhos, Pedro Leopoldo, and Capim Branca) in 2021 (Figure 4b). In the scanning analysis of the case fatality rate among those hospitalized for severe COVID-19, six local clusters were detected, with 36 municipalities having a higher risk of death (RR) (*p* ≤ 0.05) among those hospitalized compared with other municipalities in the MRBH. Clusters with RRs of 2.42 (Itabirito and Rio Acima), 2.28 (Mário Campo and Sarzedo), 2.09 (13 municipalities in the Sete Lagoas microregion), 1.85 (8 municipalities in the Betim and Itaúna microregions), 1.50 (10 municipalities in the Belo Horizonte and Itabira microregions) and 1.40 (Ribeirão das Neves) were observed (Figure 4c).

## 4. Discussion

The implementation of a Public Health Emergency Response Plan [15] required an efficient reorganization of the healthcare network to meet the high demand for clinical and intensive care hospital beds in the fight against the COVID-19 pandemic. The significant increase in the need for hospitalizations represented a major challenge for healthcare systems. The crisis also highlighted the vulnerability of the population, social inequalities [16], and difficulties in access to healthcare. This study describes the patterns of severe COVID-19 occurrence in the MRBH (the third most populous region in Brazil) from 2020 to 2022, illustrating that, although the risk of hospitalization was higher in the central region, the risk of death was higher in areas that were more peripheral.

The municipalities in the central area of the MRBH, especially the most populous ones with better infrastructure [10], recorded higher numbers of hospitalizations, hospitalization rates, and a higher absolute number of deaths from severe COVID-19 in the study period. However, the distribution pattern of the mortality and case fatality rates among hospitalized patients did not necessarily follow that observed for the risk of hospitalization, especially in regard to the formation of clusters with higher numbers of hospitalizations and deaths. Only in 2020 was the mortality rate higher in the more central municipalities. In the other two years, there was no distribution pattern for the mortality rate. In contrast, the case fatality rate among hospitalized patients followed a peripheral distribution pattern in municipalities with a less structured public health network.

During the study period, the highest rates of hospitalization due to severe COVID-19 were concentrated in the central region of the MRBH, whereas the most peripheral municipalities of the MRBH had lower numbers of hospitalizations due to severe COVID-19 at the beginning of the pandemic. Municipalities in the central area of the MRBH had a higher risk of hospitalization among their local residents in the early months of 2021, a period representing the beginning of vaccination against COVID-19 [12]. After this period, no municipality presented a high risk of hospitalization. An increase was observed in the number of hospital beds in the municipalities with the highest hospitalization rates, such as Belo Horizonte and Contagem, which had the largest increases in 2020. Belo Horizonte increased the number of hospital beds in 2021 [9], which was a period when there was a high rate of hospitalization and a predominance of the Alpha variant [12]. In general, reductions or smaller increases in the number of hospital beds occurred during the period in which vaccination began in 2021 [12]. Other municipalities also increased the supply of hospital beds in 2021 [9] when the second wave occurred and there was a high demand for hospital beds and increased mortality due to the Gamma variant [12]. In 2022 [9], there was another increase in the number of hospital beds when the third wave occurred; this period was characterized by lower mortality and higher morbidity due to the emergence of the Omicron variant [12].

While our analyses were conducted using annual data (2020, 2021, and 2022), it is important to highlight that each of these years was characterized by the predominance of different SARS-CoV-2 variants in Brazil. In the MRBH, as in other regions of the country, the Alpha variant predominated in early 2021, followed by the Gamma variant during the second wave, while the Omicron variant became dominant in early 2022 [12]. Although we did not stratify our results by variant or viral lineage, the differences observed in hospitalization and mortality rates over time may partially reflect the distinct characteristics of each wave, including differences in transmissibility and virulence, as well as the introduction of vaccination.

Municipalities with higher hospitalization rates tend to have access to health actions and services [17], enabling higher levels of diagnosis and hospitalization [9]. These municipalities also exhibit better healthcare structures [10]. A study carried out in three municipalities in Brazil showed differences between the municipalities in their response actions during the pandemic [18].

The highest mortality rate was found in residents of municipalities with greater social vulnerability (Betim, Ibirité, Mário Campos, Sabará, and Raposos in 2020 and in Moeda in 2022). A higher risk of mortality was also observed in the microregional municipalities of Sete Lagoas and Vespasiano. Sete Lagoas and Betim are microregion hubs and have higher numbers of hospital beds [9]. The other municipalities have less developed health infrastructure and more socially vulnerable populations [10]. This corroborates a prior study that showed higher mortality in more vulnerable regions [19,20,21,22]. These findings highlight the need for a health plan aimed at expanding health access to more vulnerable populations. One study in Brazil showed that inequalities affected the course of the pandemic and that policies are needed to protect people with greater socioeconomic vulnerability [23]. Our study found a higher risk of case fatality due to severe COVID-19 among hospitalized patients in residents of eight peripheral municipalities of the MRBH, encompassing the Sete Lagoas and Vespasiano health microregions [6] during a period in which there was an increase in deaths related to the Alpha, Gamma, and Omicron variants [12]. The case fatality rate among hospitalized patients was higher among residents of municipalities with greater social vulnerability (Rio Acima, Itabirito, Sarzedo, and Mário Campos), as well as in thirteen municipalities of the Sete Lagoas, Betim, Pará de Minas, and Vespasiano health microregions [6]. This finding is also corroborated by other studies conducted in Brazilian cities. In the Northeast of Brazil, municipalities with greater social vulnerability exhibited higher case fatality rates among hospitalized patients [20]. Another study showed higher mortality rates in the North and Northeast of the country in municipalities in the countryside, and some patients who died had not been hospitalized [24]. The prevention and control measures adopted in each state of the Northeast region of Brazil were different during the pandemic and impacted the number of deaths and the morbidity and mortality of the elderly residents of these regions [25]. The municipalities of Pará de Minas, Nova Lima, and Sete Lagoas [6] are hubs of health microregions and had high case fatality rates among hospitalized patients, demonstrating the need to strengthen surveillance actions and the management of public health emergency actions, such as planning effectiveness [26].

In contrast, the hospitalized patient mortality rate was lower in the central region of the MRBH, where preventive measures had a greater impact. Belo Horizonte demonstrated the ability to respond quickly to PHEs according to the needs of the population and was able to plan and organize the health system response through the provision of services. These data show how the greater capacity to respond to a PHE influenced the outcome in the central area of the MRBH [27].

One study carried out in the state of São Paulo during the COVID-19 pandemic highlighted the need for municipal actions to strengthen the healthcare network, such as opening new intensive care units (ICUs) and infirmary beds, reorganizing existing hospital beds, and regionalizing healthcare [28]. Other studies have shown that having access to beds and health services resulted in lower mortality rates [29,30]. Health surveillance provides information that allows managers to assess the need to adopt new management strategies [1].

In the MRBH, it was observed that the increase in the number of private hospital beds was larger than that of public hospital beds, even in Belo Horizonte, where the largest number of hospital beds was made available during the pandemic [9]. One study even illustrated that there were fewer public hospital ICU beds than private ones, indicating an inequality in access to hospital beds [31]. Such states as Maranhão, Rio Grande do Sul, and Espírito Santo, as well as cities like Curitiba and São Paulo, reorganized the health network in partnership with the Unified Health System (SUS) and the private sector [31]. The supply of hospital beds in Brazil during the COVID-19 pandemic showed that regionalization is a form of organization in routine situations, but that the large displacement of patients to access health services compromised the health and lives of patients with more serious disease [32].

The differences in the performance of managers and in the organization of health networks resulted in varied outcomes among municipalities. Service organizations that operate in routine situations have proven to be inefficient in public health emergencies [32]. This study provides evidence of the need for an efficient Public Health Emergency Response Plan that supports surveillance and response actions and is capable of meeting the actual demands of the population. The distribution of hospitalizations and deaths due to severe COVID-19 in the MRBH presented distinct spatial and spatiotemporal patterns that reflect the structural health inequalities between municipalities. While municipalities in the central area, such as Belo Horizonte, had the largest number of hospitalizations, peripheral municipalities showed higher mortality and fatality rates due to severe COVID-19. These findings suggest the need for specific and regional preparation and responses to mitigate the impacts of PHEs. Disparities in access to health and service capacities in more vulnerable regions require more agile actions by managers to increase the number of hospital beds so as to reduce mortality rates. This study has certain limitations, including the potential for differences in data completeness, missing data, and data quality due to the system’s reliance on notification records and the database management practices employed by various municipalities, which have different infrastructure and technical capacities, as well as the exclusion of hospital admission waiting times, hospital bed occupancy rates, and individual-level factors such as age, comorbidities, and vaccination status, which could provide additional context for interpreting the observed spatiotemporal patterns. Furthermore, our analysis was not stratified by specific SARS-CoV-2 variants and assessment of the potential differential impacts of variant waves on the mortality and case fatality rates among hospitalized patients was not feasible. Another limitation concerns the lack of information distinguishing between public and private healthcare services, as differences in infrastructure, such as ICU bed availability, care protocols, and access to timely treatment, across these sectors may have influenced hospitalization and mortality outcomes but could not be explored due to data constraints. Finally, as an ecological study based on aggregated data, our findings cannot be interpreted as causal relationships at the individual level.

## 5. Conclusions

The spatiotemporal disparities in hospitalization and mortality rates revealed health inequities that may be influenced by social determinants of health and structural vulnerabilities within healthcare systems. These patterns reflect differential exposure to risk factors, unequal access to quality healthcare services, and varying capacity for disease prevention and management across different populations and geographic areas. It was possible to identify priority areas that require special attention in the regional planning of the care network, especially with regard to referral and counter-referral flows. In non-PHE periods, these flows operate with known limitations; however, in PHE contexts, such as pandemics, these weaknesses intensify and contribute to the unequal healthcare impacts between territories. It is therefore essential that planning be carried out in advance and based on evidence, such as that presented herein, allowing the reorganization of care flows and the allocation of resources to occur in a timely manner. This preparation must be conducted in an interconnected manner, i.e., integrating the municipal and state levels, with support from the federal government, and must involve intersectoral strategies that consider the social determinants of health and the complexities of regional networks in order to ensure a more equitable and efficient response to future PHEs. Our study contributes to a critical reflection of the organization of the health system by revealing inequalities in health outcomes that are linked to healthcare capacity and local vulnerabilities.

## Data Availability

The data were made available by the State Health Department of Minas Gerais, Brazil. The data are nominal and may be made available by the authors upon request.

## Abbreviations

IBGE	Brazilian Institute of Geography and Statistics
ICU	Intensive care unit
MRBH	Metropolitan Region of Belo Horizonte
PHEs	Public Health Emergencies
SARS	Severe Acute Respiratory Syndrome
SIVEP-GRIPE	Influenza Epidemiological Surveillance Information System
SUS	Unified Health System
